# Iron Metabolism in Normal and Pathological Pregnancies and Fetal Consequences

**DOI:** 10.3390/metabo12020129

**Published:** 2022-01-29

**Authors:** Charles Mégier, Katell Peoc’h, Vincent Puy, Anne-Gaël Cordier

**Affiliations:** 1 Assistance Publique-Hôpitaux de Paris, Service de Gynécologie-Obstétrique, Hôpital Bicêtre, Université Paris Saclay, 94270 Le Kremlin-Bicetre, France; charles.megier@aphp.fr; 2 Assistance Publique-Hôpitaux de Paris, Laboratoire de Biochimie Clinique, HUPNVS, Hôpital Beaujon, Clichy and Université de Paris, UFR de Médecine Xavier Bichat, INSERM U1149, F-75018 Paris, France; katell.peoch@aphp.fr; 3 Unité de biologie de la Reproduction CECOS, Hôpital Antoine Béclère, Université Paris Saclay, 92140 Clamart, France; vincent.puy@aphp.fr; 4 Laboratoire de Développement des Gonades, UMRE008 Stabilité Génétique Cellules Souches et Radiations, Université de Paris, Université Paris-Saclay, CEA, F-92265 Fontenay-aux-Roses, France; 5 INSERM, 3PHM, UMR-S1139, F-75006 Paris, France; 6 PremUp Foundation, F-75014 Paris, France; 7 Assistance Publique-Hôpitaux de Paris, Service de Gynécologie Obstétrique, Hôpital Antoine Béclère, Université Paris-Saclay, 92140 Clamart, France

**Keywords:** iron, placenta, pregnancy, hepcidin, heme, globin, preeclampsia, hemoglobin

## Abstract

Iron is required for energy production, DNA synthesis, and cell proliferation, mainly as a component of the prosthetic group in hemoproteins and as part of iron-sulfur clusters. Iron is also a critical component of hemoglobin and plays an important role in oxygen delivery. Imbalances in iron metabolism negatively affect these vital functions. As the crucial barrier between the fetus and the mother, the placenta plays a pivotal role in iron metabolism during pregnancy. Iron deficiency affects 1.2 billion individuals worldwide. Pregnant women are at high risk of developing or worsening iron deficiency. On the contrary, in frequent hemoglobin diseases, such as sickle-cell disease and thalassemia, iron overload is observed. Both iron deficiency and iron overload can affect neonatal development. This review aims to provide an update on our current knowledge on iron and heme metabolism in normal and pathological pregnancies. The main molecular actors in human placental iron metabolism are described, focusing on the impact of iron deficiency and hemoglobin diseases on the placenta, together with normal metabolism. Then, we discuss data concerning iron metabolism in frequent pathological pregnancies to complete the picture, focusing on the most frequent diseases.

## 1. Introduction

Iron is an essential micronutrient that plays a critical role in dioxygen transport, storage, and delivery through hemoglobin (Hb) and myoglobin. Iron is also required for cellular respiration, energy production, DNA synthesis, and cell proliferation, mainly as a prosthetic group in hemoproteins and as part of iron-sulfur clusters [1]. However, excess iron generates reactive oxygen species through the Fenton reaction [2], leading to cellular and tissue injury [3,4]. This can ultimately lead to cell death by ferroptosis [5] or iron-dependent apoptosis [6]. Both excess iron and iron deficiency can be observed in humans.

Iron deficiency is the most common nutritional deficiency worldwide [7,8]. Women are at high risk of developing iron deficiency, and possibly anemia, particularly during pregnancy, and iron supplementation is almost universally recommended in this setting. Every pregnant woman should be screened for iron deficiency anemia, and treated with iron supplementation if needed (the recommended hemoglobin thresholds and iron doses for supplementation are summarized in Table 1). In addition, the American College of Gynecology and Obstetrics recommend low-dose iron supplementation in the first trimester for all women, regardless of their iron status [9]. Women with non-anemic iron deficiency should also be treated with 65 mg iron / day according to the United Kingdom guidelines [10]. 

Indeed, iron deficiency is associated with feto-maternal morbidity and mortality [11,12]. Moreover, iron plays a role in the long-term neurodevelopment of newborns and infants [13,14,15], as initially shown in rodents [16,17]. In iron overload, notably in hemoglobin diseases such as sickle-cell disease, thalassemia, and hereditary hemochromatosis, excess iron can be toxic. 

The placenta plays a crucial role in iron metabolism during pregnancy, as it is the interface between the mother and fetus. Here, we provide a narrative review of the data concerning iron and the placenta in both healthy and pathological pregnancies.

## 2. Systemic Iron Metabolism Outside of Pregnancy

Iron exists in two primary forms in the human body: heme, and non-heme iron. Heme iron is ferrous (Fe^2+^), whereas non-heme iron can be ferrous or ferric (Fe^3+^). Heme is a complex of protoporphyrin IX and iron. Although heme iron represents a minor proportion of dietary iron, its high bioavailability makes it a crucial iron source [18]. Such a high bioavailability implies the existence of a specific intestinal import pathway.

**Figure 1:**
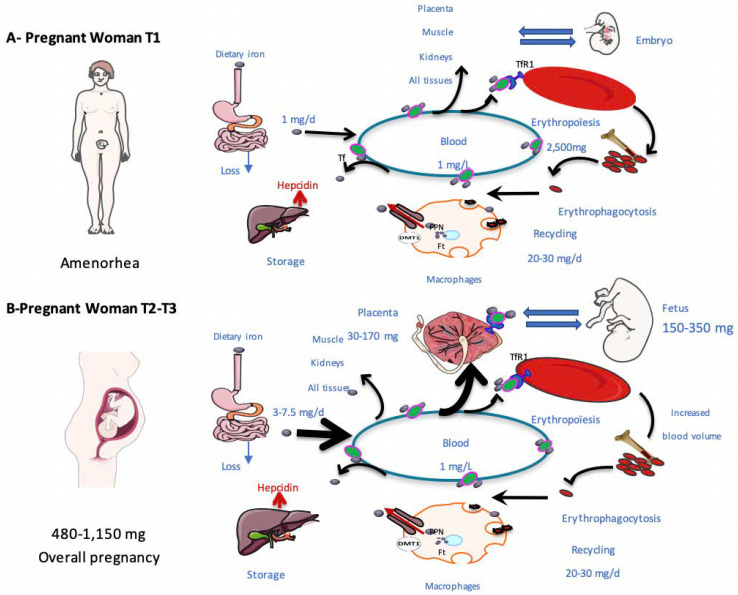
Schematic description of iron metabolism during pregnancy. This figure describes systemic iron metabolism during the first (**A**) and the second (**B**) part of pregnancy. Iron is presented as grey circles and transferrin as green circles. The average quantities of iron in the various processes are mentioned. Dietary iron is absorbed by enterocytes. Iron is distributed to various tissues and cells via transferrin in the plasma. It is internalized in tissues by endocytosis via transferrin receptors (TFR1). In pregnant women, placental TFR1 can import transferrin from the maternal circulation into the placenta through the syncytiotrophoblast. Bone marrow captures 70% of plasma iron for hemoglobin synthesis in hematopoietic precursors. At the end of their life, erythrocytes are phagocytosed by macrophages. The liver plays a significant role in iron storage. Hepcidin is synthesized by the liver and can contribute to the internalization and degradation of FPN at the basal side of enterocytes and on the macrophage membrane. During the second part of the pregnancy, the iron need increased. Tf: transferrin, TFR1: transferrin receptor 1, FPN: ferroportin.

The main features of iron metabolism are summarized in Figure 1. Enterocytes absorb 15% of heme iron and 85% of non-heme iron, non-heme iron mainly coming from plant-based food [19,20]. Iron absorption can be modified by dietary components, such as ascorbic acid and polyphenols [21,22]. At the apical side of enterocytes, Fe^3+^ is reduced to Fe^2+^ by duodenal cytochrome B (DCYTB) [23,24,25] and carried into the cells by divalent metal transporter 1 (DMT1 = SLC11A2) [26]. Once in enterocytes, Fe^2+^ can be incorporated into ferritin and stored. Ferritin represents the main iron storage protein, and one molecule can carry up to 4,500 Fe ions after oxidization [27]. Alternatively, iron can be exported to the blood circulation through the iron exporter ferroportin (SLC40A1, FPN) [28,29]. FPN lies on the basal side of intestinal cells. In blood vessels, Fe^2+^ needs to be oxidized into Fe^3+^ by a ferroxidase to be transported by the protein transferrin (TF), the principal iron transporter in the organism [26,30]. 

Animal-based food is the primary source of heme iron [20]. Heme iron is carried from the gut lumen into enterocytes by heme carrier protein 1 (HCP1), a transporter located on the apical side of the cells [31]. HCP1’s precise role is still debated, as it was more recently also identified as a folate transporter [32]. Another transporter, called HRG-1 protein (heme-responsive gene 1 protein), is suspected to be involved in heme-iron transport [33,34]. Heme can be catabolized by heme oxygenase (HO) into biliverdin, carbon monoxide (CO), and iron [35]. Once imported in the enterocyte, heme iron can be transported to the blood circulation via feline leukemia virus subgroup C receptor-related protein 1 (FLVCR1, on the basal side of enterocytes) [21,36,37].

One or two Fe^3+^ atoms can be bound by one transferrin molecule, forming holo-transferrin. Transferrin saturation (TSAT) is the ratio of serum iron to total iron-binding capacity. TSAT is an important biochemical marker of iron status, less affected by inflammation than ferritin [38]. Under normal conditions, transferrin is saturated at a third of its capacity. TSAT fluctuates with the dietary intake of iron, inflammation, chronic disease, glomerular disease, hormonal variations, and hepatocellular damage, among other factors [39].

Holo-transferrin is internalized by clathrin-mediated endocytosis through the transferrin receptor 1 (TfR1) of target cells. Acidification inside endosomes releases iron from transferrin, and transferrin and TfR1 are recycled. Fe^3+^ is reduced to Fe^2+^ by a ferrireductase, notably six-transmembrane epithelial antigen of the prostate 3 (STEAP3), before being exported to the cytoplasm by endosomal DMT1 [40]. 

Hepcidin is the sole known regulator of systemic iron concentration [41]. It acts as a negative regulator of iron absorption and reuse, leading to decreased circulating iron concentrations (it is an hyposideremic factor). This peptide is synthesized in the liver and rapidly excreted via the kidneys [42]. Hepcidin decreases intestinal iron absorption via its interaction with FPN through its internalization and degradation [43,44]. Moreover, hepcidin restricts iron export from macrophages through internalization and degradation of membrane FPN to regulate iron recycling [43,44,45]. Thus, iron is trapped in cells and the serum iron concentration decreases. Hepcidin is upregulated by inflammation and systemic and tissue iron content and decreased by erythropoietic demand and hypoxia [46,47]. 

Most iron recycling occurs through intra-tissular and intravascular hemolysis (20 and 1–2 mg/day, respectively) [30]. Intra-tissular hemolysis or erythrophagocytosis consists of the phagocytosis of old erythrocytes at the end of their lifespan (120 days) and represents 80 to 90% of total hemolysis. It occurs in macrophages in the liver, spleen, bone marrow, and reticuloendothelial system. After the phagocytosis of senescent erythrocytes, hemoglobin is catabolized with, notably, the catabolism of heme by heme oxygenase (HO) [48]. Intravascular hemolysis, representing 10 to 20% of total hemolysis, produces hemoglobin and heme. Hemoglobin is bound to haptoglobin, and the hemoglobin-haptoglobin complex can be internalized by macrophages through their receptor CD63 [48]. Heme from intravascular hemolysis can end up in plasma, where it is complexed to hemopexin [48]. Heme-hemopexin complexes can be internalized by cells, notably macrophages, that express their receptor, called CD191. After endocytosis, heme is broken down by HO [49], allowing iron recycling.

## 3. Adaptation to Physiological Pregnancy

### 3.1. Iron Needs during Pregnancy

The average iron cost of pregnancy in human varies between 480–1150 mg. In addition to the mother’s needs, 30–170 mg and 200–450 mg of iron are required for both placental and fetal needs, respectively [50]. The maternal plasma volume and red blood cell (RBC) mass increase by 30 to 50% and 20 to 30% throughout pregnancy, respectively. At the same time, the RBC life span decreases slightly (~9%) and erythropoietin levels increase (doubling by the end of the third trimester) [51]. As a result, the mother’s iron needs increase throughout pregnancy.

Approximately 1 mg of daily iron intake is required during the first trimester of pregnancy [51]. This is lower than for a non-pregnant woman because of the absence of menstruation. In the third trimester, physiological requirements vary between 3 to 7.5 mg/day, representing a mean increase of 4.1 mg/day above median pre-pregnancy needs [51]. During the last trimester, the iron concentration in the body of the fetus is approximately 75 mg per kilogram [52], with most fetal iron being stored in RBCs (70%). Consequently, mothers are frequently proposed iron supplementation to prevent deficiency, particularly in iterative or multiple pregnancies.

Changes in some iron parameters during un-supplemented pregnancies are presented in Table 2.

### 3.2. Adaptation of Iron Metabolism to Pregnancy

Both dietary iron absorption and iron mobilization from storage are enhanced during pregnancy to meet increasing needs. This concerns both heme and non-heme iron [53,54,55]. The hepcidin concentration decreases throughout normal pregnancy and its nadir is usually reached during the third trimester, as the iron needs are maximal [41,56]. The exact pathways involved in hepcidin regulation during pregnancy are unknown. In rodent models of pregnancy, DMT1 and DCYTB levels increase, with a decrease in maternal hepcidin concentrations [57].

This decrease may be in response to the rise in fetal and maternal iron demand, notably for erythropoiesis. The very mild inflammation that occurs in healthy pregnancies does not increase hepcidin concentration sufficiently to counterbalance its physiological decrease [58]. Hepcidin mRNA expression is lower in the livers of fetuses from dams fed iron-deficient diets, showing that hepcidin is expressed during development and responds to the environment [59,60]. However, the exact role of fetal hepcidin is yet to be unraveled. Recent data has shown that fetal liver hepcidin participates in building hepatic iron storage in the third trimester of gestation but that placental FPN is not regulated by fetal hepcidin, unlike liver FPN [61]. 

### 3.3. Sources of Placental Iron

**Figure 2:**
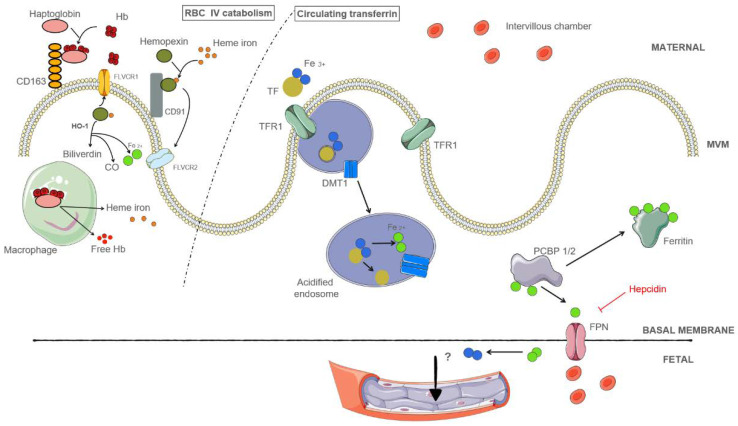
Main features of iron transport and metabolism in the placenta [26,37,48,62,63]. This figure describes placental iron import from maternal holo-transferrin, which is the main origin of iron, and intravascular hemolysis. Briefly, iron can be internalized in the heme form, as hemoglobin or heme, complexed with either haptoglobin or hemopexin, respectively, through their receptors CD163 and CD91. Heme iron is degraded by HO and the resulting free iron can be either stored or exported. Internalization by clathrin-mediated endocytosis of the complex of transferrin and its receptor leads, after the formation of an acidic endosome, to the export of reduced iron into the cytoplasm, where it can be stored or exported. FLVCR1: feline leukemia virus subgroup C receptor-related protein 2, FLVCR2: feline leukemia virus subgroup C receptor-related protein 2, HO-1: heme-oxygenase 1, Fe: iron, Hb: hemoglobin, TF: transferrin, TFR1: transferrin receptor 1, DMT1: divalent metal transporter 1, poly(rC) binding protein (PCBP) 1 and 2, VM: villosity membrane, FPN: ferroportin.

During pregnancy, substantial amounts of iron are exchanged across the placenta, and the iron needs of the fetus must be matched by the transplacental transport of maternal iron. The main source of placental iron is maternal holo-transferrin. Maternal intravascular hemolysis may also be involved, depending on the iron stores but this hypothesis needs to be confirmed by further studies (Figure 2).

#### 3.3.1. Placental Iron Is Obtained from Holo-Transferrin in the Maternal Circulation 

Holo-transferrin in the maternal circulation can be imported into the placenta through the syncytiotrophoblast (ST) by placental TFR1 [62,64]. Transferrin is essential for iron regulation and fetal development. Indeed, TFR1 knockout in mice causes primary anemia and death before the twelfth day of gestation [65]. TFR1 is highly expressed on the apical side of the ST and the complex is internalized by endocytosis [66]. Once imported into the ST, Fe^3+^ dissociates from transferrin in the acidic endosomes. Fe^3+^ is reduced to Fe^2+^ by ferric reductases and exported into the ST cytoplasm by placental DMT1 [26,62]. DMT1 is ubiquitously expressed in embryonic and placental tissues in the first trimester [67]. Its placental expression during the third trimester rises in maternal anemia [68]. Aside from DMT1, ZIP8 (SLC39A8) and 14 (SLC39A14) may also be involved in iron export from the endosome to the ST cytoplasm. They are both Fe ^2+^ exporters and located in the human placenta [69,70]. ZIP14 expression is lower than that of ZIP8, and ZIP14 knocked-out mice are viable [71].

ZIP8 expression increases during pregnancy in rodent models and its knockout is responsible for embryonic death [70]. In the ST cytoplasm, iron can be either carried into the fetal circulation by placental FPN/DMT1 or stored as ferritin. The multifunctional adaptor proteins poly(rC)-binding protein (PCBP1 or PCBP2) could be responsible for delivering Fe^2+^ to either FPN or ferritin [26,62]. Both heavy- and light-chain ferritin gene expression (FTH1 and FTL) are upregulated in almost all placental cell types (especially trophoblastic and decidual cells), participating in the constitution of the placental iron supply during pregnancy [72].

How iron is transported through the ST basal membrane into the fetal endothelium is uncertain. FPN and DMT1 may be involved, as they are located at the junction of the ST basal membrane and fetal vessels [73,74]. In mice, FPN is required for embryogenesis; disrupting its gene leads to embryonic death [28]. There is currently no data on how iron is transported across the fetal endothelium [75,76]. Ferroxidases, such as ceruloplasmin and hephaestin, located in the placenta, may oxidize Fe^2+^ into Fe^3+^ to permit iron transfer to fetal transferrin, but whether this actually occurs is still unclear [62,77]. 

#### 3.3.2. Placental Iron May Be Obtained from Hemolysis

Maternal intravascular hemolysis represents 10% to 20% of total hemolysis and produces hemoglobin and heme. Hemoglobin is bound to haptoglobin and the hemoglobin-haptoglobin complex can be carried into the ST through CD163 [26,71]. CD163 is expressed in the human placenta and on macrophage-like cells of the chorion and decidua.

Circulating heme molecules form complexes with hemopexin and the heme-hemopexin complex can be carried into the ST by CD91 [26,78]. It has been suggested that heme iron may also be imported into trophoblastic cells by FLVCR2, a heme importer, whereas FLVCR1 may work as a heme exporter. Further studies are needed to support these assumptions [37].

### 3.4. Regulation of Placental Iron Transport

Placental iron import is regulated by iron regulatory proteins (IRPs) 1 and 2 [79,80]. These proteins have a regulatory function on molecules involved in iron metabolism, such as TFR1, FPN, and ferritin. IRPs regulate the expression of many genes involved in iron metabolism by binding to mRNA stem-loop structures, called iron responsive elements (IREs), localized at either the mRNA untranslated 5’ or 3’ regions. Intracellular iron, which can bind to apo-IRP, modulates IRP activity through distinct mechanisms [81]. The recognition of an IRE located in the 5’-untranslated region by an IRP inhibits activation of the mRNA translation initiation complex and therefore the translation of the mRNA [82]. Conversely, the recognition of an IRE in the 3’-untranslated region by an IRP prevents degradation of the mRNA.

### 3.5. The role of Heme Oxygenase during Pregnancy

HO provides an iron source by catabolizing free heme into biliverdin, CO, and iron [83,84]. Transcripts, including the transcript encoding the inducible HO-1 isoform *HMOX1* and the constitutive form *HMOX2*, are detected in chorionic villi, the chorionic plate, the basal plate, fetal membranes, the ST, the endothelium, and smooth muscle cells. HOs are necessary for a healthy pregnancy and normal development at various stages, including placental implantation and maintenance, spiral artery remodeling, tolerogenic allograft/host environment [84,85]. HOs are highly expressed during pregnancy and their overall expression is lower in pathological human pregnancies [84]. A number of studies have shown that HO mRNA levels increase during gestation [86]. In *HMOX1*^-/-^ mice, miscarriages and fetal loss are frequent, with decreased survival [87]. *HMOX1* transcripts have been shown to regulate angiogenic factors, such as soluble vascular endothelial growth factor receptor-1 (SFLT1) and placental growth factor (PLGF) in pathological pregnancies [88]. The importance of HOs in pregnancy highlights the role of heme iron.

### 3.6. Exploration of Iron Metabolism during Pregnancy

Screening for iron deficiency should be considered before anemia occurs, as it occurs prior to anemia. International guidelines agree that all pregnant woman should be screened for anemia using a complete blood count during the first trimester and at 28 weeks of gestation (WG) [10,89,90]. A Hb concentration below 11 g/dL defines anemia during pregnancy. The Hb concentration declines during the first trimester, reaches its nadir during the second trimester, and rises again during the third trimester [91]. Although the WHO does not recommend trimester-specific thresholds for anemia diagnosis, it states that the Hb concentration physiologically diminishes by approximately 0.5 g/dL during the second trimester [91]. 

The intracellular iron storage pool correlates with the circulating ferritin concentration [92]. Without iron supplementation, the ferritin concentration decreases throughout pregnancy, with a nadir between 35 and 38 WG, and then slowly increases afterward [54]. Serum ferritin < 30 µg/L is the main criterion for iron deficiency for pregnant women if no chronic inflammation is suspected. This threshold shows 90% sensitivity and 85% specificity for the diagnosis of iron deficiency during pregnancy without iron supplementation [93].

Most commercially available methods are based on immunochemistry assays. Apoferritin is comprised of 24 subunits of two types, (heavy (H) and light (L), assembled in varying proportions. H and L subunits are encoded by distinct genes and have different non-redundant functions: H subunits oxidize Fe^2 +^ to Fe^3 +^ and allow its entry into the molecule; L subunits facilitate the formation of iron crystals. Thus, variable proportions of H and L subunits may affect the intracellular Fe^2+^/Fe^3+^ ratio. The measurement of circulating ferritin may vary and is still imperfectly standardized. Notably, the balance of anti-H and anti-L antibodies used in different commercial immunoassays varies, contributing to the explanation of the observed variation. A higher plasma volume and modifications in the cytokine profile can also interfere with measurement of the ferritin concentration. 

TSAT and the mean corpuscular volume (MCV) are frequently used to assess iron deficiency in pregnant women [38,94]. The MCV is a non-specific marker, as it is distorted by the high number of erythrocytes due to the increase in erythropoiesis [38,93]. On the other hand, TSAT can be affected by food and circadian variations, but is more informative in an inflammatory environment, after adaptation for the gestational age. TSAT, ferritin, soluble TFR, Hb, hematocrit, MCV, erythrocytes, and reticulocyte counts should be used to provide an exhaustive assessment of the iron status if the situation is not sufficiently clear-cut.

## 4. Iron Metabolism in Pathological Pregnancies

### 4.1. Preeclampsia

Preeclampsia (PE) is a pregnancy disorder defined by hypertension and proteinuria, associated with a pro-inflammatory state and hypoxia [95,96]. It complicates 2% to 5% of pregnancies and is one of the leading causes of maternal death in developed countries [97]. With delivery before 34 WG, early-onset PE represents 42% of all PE cases and appears to be caused by abnormal trophoblastic invasion and remodeling of placental spiral arteries [98]. Instead, late-onset PE (with delivery after 34 WG) represents 58% of PE cases and is likely induced by fetal demands exceeding the placental capacity [98]. Both situations result in endothelial dysfunction, which leads to hypoxia. Iron metabolism is disturbed during PE, but differently depending on the term at which it occurs. Hepcidin levels are increased by inflammation and decreased by hypoxia, of which both occur during PE. 

#### 4.1.1. Early-Onset Preeclampsia (<34 WG)

In a case-control study comparing women with third trimester PE and pregnant controls, the serum iron concentration was higher in the women with PE (161.3 µg/L [117.20–194.70] versus 116.8 µg/L [82.45–145.74], *p* = 0.012) [99]. Similarly, the serum ferritin concentration, hematocrit, and TSAT were also higher in the women with PE than controls. At 31 weeks of gestation, the mothers with PE had lower serum hepcidin concentrations than those without (46.5 versus 51.5 ng/mL, *p* = 0.047) [99]. This is surprising, as women with PE showed higher levels of pro-inflammatory cytokines, such as interleukin-6 and tumor necrosis factor-α (IL-6 and TNF-α), than controls, suggesting that this observation results from multiple events.

The higher iron serum concentration in early-onset PE may participate in downregulating TFR1 and be related to alterations of its expression in placental tissue [100]. Such negative feedback may involve modifications in the TFR1 glycosylation sites. A recent study examined the placenta from different populations of pregnant women (controls, women with iron-deficiency anemia, and women with severe early-onset PE, six per group) who underwent an elective cesarean at 39, 38, and 31 WG, respectively [101]. The glycosylation profiles of TFR1 were different for the women with PE from those of the control and iron-deficiency anemia groups. Gal-GlcNAc and mannose patterns of TFR1 in trophoblastic cells varied between groups. The glycosylation of TFR1 binding sites could be responsible for the failure of TfR1 export to the ST apical membrane or the decrease in binding. The decrease in TFR1 expression or the glycosylation of its binding sites may be associated with high serum iron concentrations, but this has yet to be proven. 

Carbon monoxide (CO), produced by the catabolism of heme by HO, is reduced during PE pregnancies. In a study of Baum et al., 22 patients with pregnancy hypertension or PE were compared to 20 controls [102]. Blood CO concentrations after 31 WG were lower for the women with hypertensive disorders than in the control group (1.17 ± 0.35 versus 1.70 ± 0.54 ppm, *p* < 0.01). Moreover, women with PE exhaled less CO than women with healthy pregnancies [103], compatible with a decrease in HO activity. Smoking appeared to confer protection against PE, and CO concentrations were higher for smokers [88,104,105].

#### 4.1.2. Late-Onset Preeclampsia (≥34 WG)

A meta-analysis confirmed that, as in early-onset PE, women with late-onset PE have higher serum iron concentrations than women without PE (SMD = 0.28, 95%CI [0.11–0.44], *p* for Z test = I2 = 97.4%). An analysis stratified by geographic origin showed that serum iron concentrations were higher in patients with PE than healthy controls in Asian and European populations [106]. 

In a study by Shaji Geetha et al., such a difference in serum iron concentration between PE and healthy patients (5.880 versus 3.900 µg/L, *p* < 0.001) was observed at 34 WG [107]. Higher serum iron concentrations were associated with higher TSAT (76 versus 35%, *p* < 0.001). The authors also observed higher serum hepcidin concentrations in PE patients (0.684 versus 0.588 ng/mL, *p* < 0.001) than controls [107]. They found a positive correlation between the hepcidin concentration and markers of endothelial dysfunction and oxidative stress. The two groups of patients received systematic iron supplementation from the first trimester (under 200 mg of daily ferrous sulfate iron supplementation). This could explain the unusually high concentrations of iron and low concentrations of hepcidin. We can thus only interpret the tendency observed between the two groups.

In a study by Cardaropoli et al. [108], PE patients (mean term 33 WG + 3, SD = 4.2) had mean hepcidin serum concentrations comparable to those detected in gestational age-matched non-PE controls (mean term 31 WG + 3, SD = 4.5 (46.30 ng/mL [34.48–61.2] versus 48.50 ng/mL [41.28–62.63], *p* > 0.05). There were no differences between placental hepcidin mRNA expression between PE patients and controls. On the contrary, placental hepcidin and mRNA expression were lower in PE patients without fetal growth restriction than in both the control and PE with fetal growth restriction groups. During the first half of pregnancy, maternal hepcidin concentrations were higher in patients that later developed PE than those who did not (50 ng/mL [40.19–64.09] versus. 43.6 ng/mL [33.88–55.67], *p* = 0.009).

An increase in the concentration of hepcidin and a decrease in that of TFR1 may diminish iron transport into the fetal circulation. Although they could be considered to be a protective mechanism against iron-mediated cytotoxicity, these mechanisms may also play a part in the occurrence of fetal growth restriction by diminishing fetal iron intake. 

### 4.2. The Impact of Toxoplasma and Plasmodium Infections on Iron Metabolism

Surprisingly, little is known about iron metabolism in pregnant women infected by *Toxoplasma gondii* or *Plasmodium falciparum*, two frequent parasites found worldwide, of which infections are associated with increased hemolysis. 

*Toxoplasma gondii*, an obligate intracellular parasite, can pass through the placenta and lead to severe fetal malformations during pregnancy. Pregnant women can acquire toxoplasmosis by consuming undercooked meat, vegetables, or water infected with cysts. In primary maternal infection, the risk of fetal infection increases with gestational age (2.2% at 6 WG but 56% at 30 WG) [109]. However, contamination in the third trimester leads to a smaller risk of fetal malformation. The risk of primary infection is estimated to be 0.5% during the nine months of pregnancy. With an average feto-maternal transmission rate estimated to be 30% throughout pregnancy, 1.5/1000 neonates are infected in utero [110]. INFγ plays a prominent role in the host defense against the parasite. It activates cell-mediated immunity, increases the production of ROS, degrades tryptophan, and induces noncanonical autophagy [111,112]. Rodent models have suggested that INFγ may also inhibit *Toxoplasma gondii* multiplication by limiting intracellular iron availability for the parasite, and that iron chelators could inhibit parasite growth [113]. Iron chelation inhibits *Toxoplasma gondii* multiplication, whereas iron supplementation restores the growth of the parasite. In this setting, which remains rare, further studies should be performed to evaluate the interest of iron supplementation in pregnant women.

Malaria is a blood infection caused by a protozoan of the *Plasmodium* family. Each year, 25 million pregnant women from sub-Saharan Africa are at risk of malaria, and up to 27.8% of pregnant women are infected [114].

In the general population, malaria affects iron metabolism, leading to anemia. Hemolysis and the consequent hypersplenism reduce the capacity of macrophages to recycle iron, leading to iron-deficiency anemia [115]. Peripheral parasitemia is associated with higher hepcidin concentrations mediated by inflammation, contributing to anemia [116,117]. As a result, pregnant women infected with malaria during the first trimester have a higher risk of developing anemia in the third trimester than non-infected women (Odd Ratio = 2.25 95%CI [1.11–4.55]) [118]. 

Pregnant women with a preexisting iron deficiency appear to have a reduced risk of malaria [119], although the pathways involved are uncertain [120]. The lack of iron could inhibit the growth of the parasite, and may also amplify the macrophage NO-mediated defenses against *Plasmodium falciparum* that are usually diminished by iron. Furthermore, high iron stores could enhance erythropoiesis, increasing the parasite’s virulence, as it preferentially infects the youngest erythrocytes. 

Iron supplementation was long been believed to increase the risk of malaria in pregnant women. Iron deficiency was seen as a protective mechanism to limit iron availability to the parasite [121]. A recent meta-analysis has proven such assumptions to be unfounded, as two randomized trials did not show any increase in malaria infections when pregnant women received iron supplementation [119,122,123]. However, the risk of *Plasmodium vivax* malaria was higher during the first month of iron supplementation. 

Malaria during pregnancy is associated with fetal growth restriction and infant death [116,124]. *Plasmodium falciparum* can bind to trophoblastic cells, causing placental dysfunction and placental lesions (intervillositis), which result in fetal growth restriction. The presence of *Plasmodium* in the placenta, with or without maternal anemia, has no consequence on the blood cord hemoglobin concentration or newborn iron status relative to controls [125].

## 5. Hemoglobin Diseases and Pregnancy 

### 5.1. Sickle-Cell Disease

Sickle-cell disease (SCD) is an autosomal recessive disease caused by mutations in the beta-globin genes. It increases the risk of adverse maternal and fetal outcomes during pregnancy, such as PE (Relative Risk = 2.43), maternal death, fetal growth restriction (RR = 3.72), and stillbirth (RR = 3.94) [126,127]. Anemia severity is related to the type of SCD (homozygous or compound heterozygous). SCD is associated with inflammation, with high Il-6 concentrations [128]. As Il-6 increases hepcidin transcription via signal transducer and activator of transcription 3 (STAT3) [129,130], this mechanism is likely responsible for low iron concentrations and contributes to the anemia observed during SCD [46,131].

Aroke et al. reported an extremely variable prevalence of iron-deficiency anemia during pregnancy among SCD patients, ranging from 6.7% to 83.3%, using multiple diagnostic tools (bone-marrow iron staining, serum iron levels, and/or ferritin levels) [132]. As SCD is responsible for intravascular hemolysis, SCD patients have lower concentrations of haptoglobin (0.09 versus 1.18 g/L, *p* < 0.0001) and hemopexin (0.42 versus 1.05 g/L, *p* = 0.0001) than unaffected controls [AG Cordier and K Peoc’h, Personal communication]. 

### 5.2. Thalassemia

Thalassemia is a widespread disease that consists of a total or partial lack of alpha or beta globin synthesis. On the one hand, the vast majority of patients exhibit iron overload caused by hemolysis and transfusions. They can be treated by iron chelation to decrease the impact of iron overload [133]. On the other hand, it has been shown that patients with thalassemia can also exhibit iron deficiency, which can lead to an increased risk of anemia in pregnancy [134]. However, overall, fewer women with thalassemia have iron deficiencies than those without thalassemia [135].

Thalassemia affects women’s fertility. Iron overload impairs the hypothalamic–pituitary–ovarian axis, which explains why the concentration of ferritin inversely correlates with reproductive hormone concentrations in thalassemic women [136]. Takhviji et al. thus suggested that iron chelation, especially during puberty, could be beneficial for patients with thalassemia to increase fertility [136].

## 6. Obesity, Bariatric Surgery, and Pregnancy

### 6.1. Obesity and Iron Deficiency

The prevalence of obesity has increased over the past decade, and pregnant women are also affected. In Europe, the prevalence of obesity during pregnancy is estimated to be between 7.5 and 25% [137]. Women with a pre-pregnancy body mass index > 30 kg/m^2^ have a higher risk of developing iron-deficiency anemia during pregnancy. The BMI positively correlates with the concentration of soluble transferrin receptor (sTRf) and with that of serum iron, but not ferritin [138,139]. Indeed, obesity induces chronic low-grade inflammation and an elevated CRP concentration, as enlarged adipocytes tend to secrete higher levels of pro-inflammatory cytokines, such as Il-6 [140,141,142,143]. Il-6 can increase hepcidin production by the liver, leading to intracellular iron sequestration. Furthermore, TNF-α is produced by adipose tissue and its secretion correlates with the degree of adiposity [144]. In a model of obese pregnant mice, Fisher et al. showed that excess iron promotes TNF-α production and embryotoxicity [6]. 

Although the reduction in iron storage is less severe among obese than non-obese pregnant women, iron is still less available to both the mother and placenta [139].

Obesity affects fetal iron export and is a risk factor for poor neonatal iron status. Cord blood samples of newborns born to obese women show lower serum ferritin and serum iron concentrations and TSAT than for newborns born to non-obese women [139]. The upregulation of hepcidin synthesis caused by inflammation in obese pregnant women may be responsible for these negative newborn outcomes [145].

### 6.2. Iron Status after Bariatric Surgery

After bariatric surgery, iron availability should improve, as hepcidin concentrations and the chronic inflammatory state induced by obesity drop [146]. However, women who undergo bariatric surgery appear to have a higher risk of developing anemia than those who do not [147,148]. In addition, hepcidin concentrations do not decrease after surgery [140]. Such anemia is multifactorial. First, the surgery itself is responsible for reduced stomach acid production, which leads to iron malabsorption [149]. This explains why ferritin deficiency is more frequent after bypass surgery. In addition, anatomical modifications caused by the surgery lead to vitamin B12 deficiency [149]. Patients who underwent sleeve gastrectomy, a procedure that preserves the duodenum, the leading site of iron absorption, were studied by Lefebvre et al. [140]. The concentration of sTFR decreased and serum hepcidin concentrations increased, despite reduced inflammation. DMT1 levels negatively correlated with a higher serum hepcidin concentration and FPN levels were not altered. They showed that intervention after serum hepcidin measurement was associated with improved iron utilization (increased TSAT and MCV). These data underscore the relevance of measuring serum hepcidin to define which patients require intravenous versus oral iron [140].

## 7. Long-Term Fetal Consequences of Changes in Iron Metabolism and Iron Deficiency

Iron is an indispensable element for fetal and child development. On the one hand, maternal iron-deficiency anemia undeniably affects neonatal outcomes. It is associated with preterm birth (10.2% versus 6.1%, *p* = 0.009), fetal growth restriction (1.9% versus 0.3%, *p* = 0.006) [150], and fetal distress (OR 1.23 [1.08–1.40]) [151]. Early maternal anemia is more strongly associated with low birth weight than anemia that develops in the second or third trimesters [151]. 

On the other hand, high ferritin concentrations also influence neonatal outcomes. Extensive epidemiological studies show an association between increased maternal ferritin levels and the risk of adverse outcomes, such as low birth weight, stillbirth, preterm birth (<37 WG), and neonatal asphyxia [152,153]. 

The maternal iron status can modify iron delivery to the placenta and the fetus. In a rodent model, Sangkhae et al. documented that fetal and placental hepcidin are not involved in iron import when maternal iron stores are normal or reduced [154]. Similar regulation needs to be confirmed on human placentas. In pregnant rodents with iron deficiency, placental TFR1 and DMT1 levels increase and FPN levels decrease to favor iron storage in the placental compartment rather than transferring it to the fetus. This suggests that the balance is surprisingly in favor of the mother in this model [60]. However, this paradoxical effect depended on the severity of the iron deficiency. Thus, in cases of maternal iron deficiency, it is probable that the fetus is even more likely to develop an iron deficiency.

Indeed, neonates born to an iron-deficiency anemic mother have a greater risk of developing iron deficiency during their first three months of life [155]. Maternal iron deficiency alters neurotransmitter levels and myelination and is associated with poor infant outcomes in terms of mental evaluation and psychomotor skills at five years of age [156,157].

During pregnancy, maternal iron intake has been shown to be directly associated with infant head circumference and negatively associated with birth weight and height [158]. The effect of iron supplementation on birth weight is still debated. For some authors, the risk of developing fetal growth restriction is associated with non-heme iron supplementation [158]. By contrast, others have found a higher risk of fetal growth restriction in cases of heme-iron intake [159]. 

In addition to maternal supplementation during pregnancy, delayed cord clamping and umbilical cord milking increase iron storage in newborns. According to a recent Cochrane analysis, delayed cord clamping is associated with a 30% increase in iron stores, a higher hemoglobin concentration in preterm and term newborns, and lower RBC transfusion rates in preterm newborns [160]. It also results in higher ferritin concentrations at six months of life, which is a relatively easy way to improve the iron supply in newborns.

## 8. Conclusions

Iron plays a pivotal role in normal and pathological pregnancies and affects newborn development. Iron absorption and metabolism are tightly regulated, and a full exploration of iron metabolism in pregnancy is of interest to prevent maternal and fetal complications.

This paper highlights the need for the better education of gynecologists about the necessity of the early detection of iron deficiency. Moreover, it underscores the importance of preventing or treating the deficiency to ensure the optimal development of newborns and infants.

## Figures and Tables

**Table 1:** Recommendations for iron supplementation during pregnancy [9,10]. UK: United Kingdom; ACOG: American College of Obstetrician and Gynecologist.

	UK		ACOG	
	Laboratory Thresholds	Iron Dose	Laboratory Thresholds	Iron Dose
Supplementation for anemic pregnant women	- Hemoglobin: 11 g/dL (1st trimester) - Hemoglobin: 10.5 g/dL (2nd and 3rd trimesters)	100–200 mg iron / day	- Hemoglobin: 11 g/dL (1st and 3rd trimesters) - 10.5 g/dL (2nd trimester)	Full supplementation
Supplementation for non-anemic women with iron deficiency	- Ferritin < 30 µg/L	65 mg iron / day	-	-
Systematic supplementation for all women	-	Not recommended	-	Low dose iron in the first trimester

**Table 2:** Changes in hepcidin and iron concentrations and the percentage TSAT during un-supplemented pregnancy from the graphs of van Stanten et al. [44].

	T1	T2	T3
Blood Hepcidin (nmol/L)	1.85 [1,2,3,4]	<0.5 *	<0.5 *
TSAT (%)	25 [15,16,17,18,19,20,21,22,23,24,25,26,27,28,29,30,31,32,33,34,35]	20 [14,15,16,17,18,19,20,21,22,23,24,25,26,27,28,29,30,31,32,33,34] *	10 [7.5–15] *
Blood Iron (µmol/L)	15 [11,12,13,14,15,16,17,18,19,20,21,22,23,24]	15 [11,12,13,14,15,16,17,18,19,20,21,22,23,24]	9 [8,9,10,11,12,13,14,15] *

* Statistical difference between first and second trimester, or first and third trimester. TSAT: transferrin saturation.
